# Solution of the chemical master equation by radial basis functions approximation with interface tracking

**DOI:** 10.1186/s12918-015-0210-y

**Published:** 2015-10-08

**Authors:** Ivan Kryven, Susanna Röblitz, Christof Schütte

**Affiliations:** University of Amsterdam, Science Park 904, Amsterdam, 1098 XH The Netherlands; Freie Universität Berlin, Arnimallee 6, Berlin, 14195 Germany; Zuse Institute Berlin, Takustrasse 7, Berlin, 14195 Germany

**Keywords:** CME, Adaptivity, Toggle switch, Multi-stability, Cell differentiation

## Abstract

**Background:**

The chemical master equation is the fundamental equation of stochastic chemical kinetics. This differential-difference equation describes temporal evolution of the probability density function for states of a chemical system. A state of the system, usually encoded as a vector, represents the number of entities or copy numbers of interacting species, which are changing according to a list of possible reactions. It is often the case, especially when the state vector is high-dimensional, that the number of possible states the system may occupy is too large to be handled computationally. One way to get around this problem is to consider only those states that are associated with probabilities that are greater than a certain threshold level.

**Results:**

We introduce an algorithm that significantly reduces computational resources and is especially powerful when dealing with multi-modal distributions. The algorithm is built according to two key principles. Firstly, when performing time integration, the algorithm keeps track of the subset of states with significant probabilities (essential support). Secondly, the probability distribution that solves the equation is parametrised with a small number of coefficients using collocation on Gaussian radial basis functions. The system of basis functions is chosen in such a way that the solution is approximated only on the essential support instead of the whole state space.

**Discussion:**

In order to demonstrate the effectiveness of the method, we consider four application examples: a) the self-regulating gene model, b) the 2-dimensional bistable toggle switch, c) a generalisation of the bistable switch to a 3-dimensional tristable problem, and d) a 3-dimensional cell differentiation model that, depending on parameter values, may operate in bistable or tristable modes. In all multidimensional examples the manifold containing the system states with significant probabilities undergoes drastic transformations over time. This fact makes the examples especially challenging for numerical methods.

**Conclusions:**

The proposed method is a new numerical approach permitting to approximately solve a wide range of problems that have been hard to tackle until now. A full representation of multi-dimensional distributions is recovered. The method is especially attractive when dealing with models that yield solutions of a complex structure, for instance, featuring multi-stability.

**Electronic supplementary material:**

The online version of this article (doi:10.1186/s12918-015-0210-y) contains supplementary material, which is available to authorized users.

## Background

Temporal evolution of biological systems is often driven by the interaction between different types of particles which, depending on the applications, can represent molecules, bacterias, animals, or other discrete units. In nature, in nearly every process, particle numbers are subject to random fluctuations caused by inherent stochastic noise. Simulations of such systems are usually based on Monte Carlo (MC) simulations of the underlying Markov jump processes, such as Gillespie’s famous stochastic simulation algorithm (SSA) [1]. These methods share some common disadvantages: there is always a *sampling error* that, in general, is difficult to estimate; the convergence can be quite slow too. Even computing single realisations can be quite costly if many fast reactions are present; therefore approximate MC methods like *τ*-leaping [2], averaging approaches [3, 4], and deterministic-stochastic hybrid formulations [5–7] have been introduced. The applicability of these approaches depends on the existence of a permanent timescale gap that allows to clearly distinguish between fast and slow reactions.

An alternative approach is to directly compute the probability density function (PDF) as a solution of the chemical master equation (CME). Solving the CME numerically on a large state space with a huge number of unknowns is known to be difficult [8]. Various hybrid methods were proposed to cope with the curse of dimensionality, a phenomenon that refers to the rapidly increasing number of unknowns when parametrising a multidimensional system [9–11].

However, in many cases the probability distribution has ‘significant’ values only on a very small portion of the whole state space. Here, ‘significant’ means being distinguishable from zero and refers to a value that is larger than a predefined small tolerance. This fact has motivated an exploration towards special numerical methods that exploit this feature. For example, Deuflhard et al. (two-dimensional case) [12] and Cotter et al. (three-dimensional case) [13] applied sophisticated adaptive finite element methods to solve the CME, but their approach is limited to low-dimensional problems. To cope with multi-dimensional problems, methods based on truncation of the CME to finite state space have been developed, such as the Finite State Projection (FSP) method [14–16] or the finite buffer discrete chemical master equation (dCME) [17, 18]. Based on the FSP, Kazeev et al. used Quantized Tensor Trains for a direct solution of the CME [19].

A very different approach has been taken by Wolf et al. [20], who suggested an algorithm defining a rectangular window in the state space, enclosing the essential part of the distribution that allows to perform parametrisation of the distribution with a small number of parameters. In the current paper we will try to take the latter idea even further. Firstly, we consider cases that are not restricted to one or two dimensions but try to develop a general approach. Secondly, we allow an arbitrary shape of the ‘window’ by considering a manifold that contains system states with probabilities greater than a pre-defined threshold. Thirdly, we employ the projection on Gaussian basis functions (GBF) to further reduce the computational costs.

The concept of GBF approximation has formerly been applied to various problems in polymer chemistry [21–23] and colloidal physics [24]. In this paper it is extended to the CME. To account for the fact that at a specific time point only a small part of the system states has to be considered, the system of basis functions is adapted on every time step. The idea behind the adaptation is that the unknown distribution is parametrised using only those GBFs that contribute to probability values that are significantly greater than zero. This procedure allows for a smart approximation with a very low number of approximation parameters even in the case of multi-dimensional distributions. The total number of parameters is not constant in time but changes according the distribution’s complexity. This approach also allows to capture multi-modal cases where a time-dependent process leads to splitting/merging of a few disjoint parts of the distribution. One example for such a process is the genetic toggle switch model that typically leads to multi-stable solutions [25]. Even a more comprehensive behaviour can be observed in CMEs that model cell differentiation.

Mesenchymal stem cells (MSCs) are multipotent stromal cells that can differentiate into a variety of cell types, including osteoblasts (bone cells) and chondrocytes (cartilage cells). When derived form adults, one of the applications is related to transplantation, namely either to promote regeneration of diseased or damaged tissue or to rescue defective genes [26]. Foster et al. developed a mathematical model for cell differentiation that predicts presence of multiple stable states for differentiated cells, bifurcations and switch-like transitions [27, 28]. Later, Schittler et al. expanded the model to include the progenitor state and studied the system of binary differentiation with respect to various stimuli [29]. Despite an advanced level of the mathematical description, models presented in Refs. [26, 29] recover single trajectories for the evolution of biological systems, while realistic systems of that kind are known to be composed of a whole population of cells. In theory, the transition from an ordinary differential equation model, that results in single trajectories, to a CME, that describes the evolution for the whole population of cells, is a matter of pure formalism. However, it is the complexity of algorithms one has to cope with when solving the equations numerically, that kept researchers out from the full, three-dimensional solution to the CME problem until now.

## Methods

Suppose that the evolution of *d* species interacting via *K* reaction channels is described by a Markov jump process on the state space $\Omega =\mathbb {N}^{d}$, whereby $\mathbb {N}$ denotes the set of all non-negative integers including zero. The entry $X_{i}(t) \in \mathbb {N}$ of a realisation $X(t)\in \mathbb {N}^{d}$ is the number of particles of species *i* at time *t*. Our goal is to compute the distribution *u*(*x*), *x*∈*Ω*, the probability that there are exactly *x*_*i*_ particles of the *i*^th^ species, *i*=1,…,*d*. In particular we are interested in the time dynamics of the distribution *u*(*x*,*t*),

(1) $$\begin{array}{@{}rcl@{}} & u(x,t) = \mathbb{P}\left(X(t)=x\right),\, x \in \Omega,\\ &\|u(x,t)\|=1, \, t\in [0,\infty). \end{array} $$

The distribution *u* evolves according to the CME [8]

(2) $$\begin{array}{@{}rcl@{}} \left\{ \begin{array}{c} \frac{\partial u(x,t)}{\partial t} = \mathcal{L}u(x,t)\\ u(x,0) = u_{0}(x), \end{array} \right. \end{array} $$

where $\mathcal {L}: L \rightarrow L$ denotes the operator,

(3) $${} \left(\mathcal{L}u(x,t)\right)(x)=\sum\limits_{i=1}^{K} \left(a_{i}(x-\nu_{i})u(x-\nu_{i},t) - a_{i}(x)u(x,t)\right)  $$

In Eq. (3), $\nu _{i} \in \mathbb {Z}^{d}$ denotes the stoichiometric vector that defines jumps to new states *x*+*ν*_*i*_ via the *i*^th^ reaction channel. The *x*-dependent coefficients *a*_*i*_(*x*) indicate the *i*^th^ propensity function.

Let $T_{k},T_{k}^{\text {-- }}:L \rightarrow L$ be shift operators that act along the *k*^th^ dimension of *L*,

(4) $${} \begin{aligned} \left(T_{k} u(x)\right)(x) &= u({x_{1}, x_{2},\dots, x_{k}-1,\dots},x_{d}),\quad (x_{i})\in \Omega,\\ \left(T_{k}^{-} u(x)\right)(x) &= u({x_{1}, x_{2},\dots, x_{k}+1,\dots},x_{d}),\quad (x_{i})\in \Omega.\\ \end{aligned}  $$

Let us define the *n*^*th*^-power for operators (4) as an *n*-folded composition

(5) $$\begin{array}{@{}rcl@{}} {T^{n}_{k}}&=&T^{n-1}_{k}\circ T_{k},\, n>0; \\ {T^{n}_{k}}&=&\left(T^{-}_{k} \right)^{-n},\quad n<0;\\ {T^{n}_{k}}&=&I,\, n=0, \end{array} $$

where *I* is the identity operator. Now, the CME operator (3) can be rewritten as

(6) $$ \mathcal{L}=\sum\limits_{i = 1}^{K} \left(\prod\limits_{k=1}^{d}T^{\nu_{i,k}}_{k} - I\right)W_{a_{i}}.  $$

Here, *ν*_*i*,*k*_ denotes the *k*^*th*^ component of the stoichiometric vector *ν*_*i*_, and *W*_*a*_:*L*→*L* is a multiplicative operator that takes the probability distribution *u*(*x*) to its weighted form *a*(*x*)*u*(*x*). The representation (6) is especially convenient when implementing the approximation technique.

## Results

Let *S*⊂*Ω* be a fixed and enumerated system of points $\{x^{i}\}_{i=1,\ldots,n},\; x^{i}=\left ({x_{1}^{i}},\ldots,{x_{d}^{i}}\right)$. Here the lower index denotes dimension, the upper index is a counter. Each point *x*^*i*^∈*S* has one radial basis function, *ϕ*_*i*_(*x*), associated,

(7) $$ \phi_{i}(x)=\prod\limits_{k=1}^{d} e^{-\frac{\left(x_{k}-{x^{i}_{k}}\right)^{2}}{{\sigma^{i}_{k}}}},\quad {i=1,\ldots,n},  $$

where the choice of the connectivity parameters ${\sigma ^{i}_{k}}$ is a tradeoff between coverage of the whole *Ω* and sufficiently small condition number of the following matrix,

(8) $$ \left(A\right)_{i,j} = \phi_{j}(x^{i}).  $$

The approximate solution $\tilde u(x,t)$ to the CME (3) is searched in the form

(9) $$ \tilde{u}(x,t)=\sum\limits_{i=1}^{n}\alpha_{i}(t) \phi_{i}(x), \;\boldsymbol \alpha=(\alpha_{1},\alpha_{2},\dots,\alpha_{n})^{T}.  $$

The matrix *A* is composed in such a way that the value of approximation sum (9) at points *x*^*i*^ can be simply written as a column vector: $\tilde u(x^{i},t) = A\boldsymbol \alpha $. Multiplying the last expression with *A*^−1^ on the left yields, $\boldsymbol \alpha = A^{-1}\tilde u(x^{i},t).$ Thus *A* is an interpolation matrix. It is easy to see that the CME is a linear differential equation and the discretization $\tilde {\mathcal {L}}:\mathbf {R}^{n}\rightarrow \mathbf {R}^{n}$ to the CME operator (6) can be directly used to implement a collocation scheme on nodes *x*^*i*^,

(10) $$\begin{array}{@{}rcl@{}} \tilde{u}(x,t)&=&e^{t\tilde{\mathcal{L}}}\boldsymbol \alpha_{0},\\ \boldsymbol\alpha_{0}&=&A^{-1}\left(u_{0}(x^{i})\right);\\ \tilde{\mathcal{L}}&=&\sum\limits_{i = 1}^{K} \left(\prod\limits_{k=1}^{d}\tilde{T}^{\nu_{i,k}}_{k} - I\right)\tilde{W}_{a_{i}}. \end{array} $$

Here, $\tilde {T_{k}}$ is an approximated shift operator that, together with its powers, is defined analogously to (5) using $\tilde {T},\,\tilde {T}^{--}$,

(11) $${} \begin{aligned} \tilde{T}_{k} &= A^{-1}A_{1}A,\;(A_{1})_{i,j}=\phi_{j}\left({x^{i}_{1}},{x^{i}_{2}},\ldots,{x^{i}_{k}}-1,\ldots,{x^{i}_{d}}\right), \\ \tilde{T}^{-}_{k}&=A^{-1}A_{2}A,\;(A_{2})_{i,j}=\phi_{j}\left({x^{i}_{1}},{x^{i}_{2}},\ldots,{x^{i}_{k}}+1,\ldots,{x^{i}_{d}}\right), \end{aligned}  $$

and $\tilde {W}_{a}$ is the approximation to multiplicative operator

(12) $$ \tilde{W}_{a}=A^{-1}\text{diag}\left\{a\left(x^{1}\right),a\left(x^{2}\right),\dots,a(x^{n})\right\}A.  $$

Even though. the matrix exponentiation is used in (10), there are sufficiently fast algorithms that compute a matrix exponent with up to machine precision in comparably short time, e.g. [30]. Consequently, the error is predominantly introduced by the choice of the discretization nodes *x*^*i*^.

The *essential support* is defined as those states *x*∈*Ω* for which *u*(*x*)≥0 is greater than a certain threshold,

(13) $$ \text{esupp}\{u(x)\}:= \{x:\,u(x)>p_{\text{threshold}}\}.  $$

It is often a case that the probability density *u*(*x*,*t*) at time *t* has a small essential support not only when comparing with the whole state space *Ω*, but also when comparing with the union of all essential supports over the whole period of time,

(14) $${} {\fontsize{9.4pt}{9.6pt}\selectfont{\begin{aligned} \mu\, \text{esupp}\{ u(x,t)\} \ll\mu \bigcup\limits_{t} \text{essup} \{u(x,t)\}<\mu\Omega,\quad t\in[\!0,t_{end}]. \end{aligned}}}  $$

where $\mu :\mathbb {R}^{d}\rightarrow [\!0,\infty)$ is a Lebesgue measure. Although for a fixed system of basis functions the matrix exponentiation (10) provides the exact solution avoiding time discretisation at all, the condition (14) motivates to consider the CME on sequential time steps, not as means of time approximation, but as a way of economy of computational resources. Indeed, if the system of basis functions (7) is chosen to correspond to the current location of the essential support, the total number of discretisation coefficients *α*_*i*_(*t*) will be small.

**Fig. 1:**
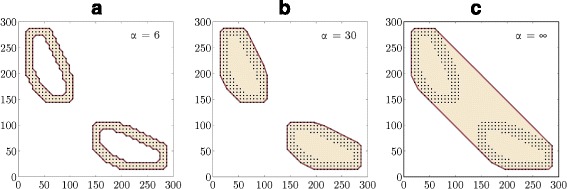
Three examples of the *α*-hull, a generalisation to the convex hull. Various values for parameter *α* yield different shapes of the domain for the same set of points: **a** two disjoint rings, **b** two disjoint disks, **c** actual convex hull

In order to associate a set of essential supports with a boundary line, the concept of the *α*-hull presented in Ref. [31] is employed. The alpha hull *S*_*α*_ is a generalisation of the convex hull of a finite set of points *S*. It provides a possibility to associate the signed distance function with a set of points as illustrated in Fig. 1. A distance operator *D*:*S*→*L* takes a set of points from *Ω* to the signed distance function

(15) $$ \left(D(S)\right)(x)=\left\{ \begin{array}{lc} -\min\limits_{y\in S_{\alpha}}|x-y|, & x \in S_{\alpha}, \\ \;\;\;\,\min\limits_{y\in S_{\alpha}}|x-y|, & x \notin S_{\alpha}, \end{array} \right.  $$

where |*x*−*y*| is an Euclidean distance. The operation (15) is reversible: by knowing a signed distance function *d*(*x*)=*D*(*S*) one may recover the set of points S,

(16) $$ S=D^{-1}d(x)=\{x:d(x)\leq0\}.  $$

Equations (15) and (16) allow applying the usual numerical toolbox, that is well defined on functions, to essential support sets. Various transformations of the signed distance function lead to changes in the set of points. For instance, let *d*_1_(*x*) and *d*_2_(*x*) be signed distance functions, then *d*(*x*)= min(*d*_1_(*x*),*d*_2_(*x*)) is the signed distance function representing the union of the interior regions [32].

Let us assume that the essential support evolves continuously in time. This means that there exists a finite vector valued function |*v*(*x*,*t*)|<*∞* that defines the normal direction speed for the essential support boundary. Moreover, *v*(*x*,*t*) also defines the evolution of the corresponding time-continuous distance function *d*(*x*,*t*) in terms of the level-set equation [32],

(17) $$ \frac{\partial d(x,t)}{\partial t}=v(x,t)|\nabla d(x,t)|,  $$

where |·| is an Euclidian norm. Suppose that the time interval [ 0,*t*_*end*_] is divided into subintervals with *k* ordered time points

(18) $$\begin{array}{@{}rcl@{}} t_{0} &=& 0,\quad t_{k} = t_{end};\\ t_{i} &<& t_{i}+1, \; t_{i} \in [\!0, t_{end}],\; i=0,1,\dots,k. \end{array} $$

As the pairwise distances between three sequential time points *t*_*i*−1_,*t*_*i*_,*t*_*i*+1_ approaches zero, the time variation of the speed *v*(*x*,*t*) vanishes as well,

(19) $$ |v(x,t_{i-2})-v(x,t_{i})|,|v(x,t_{i})-v(x,t_{i-1})|\rightarrow 0.  $$

Writing the level-set Eq. (17) for time point *t*_*i*_ using left and right finite differences to replace the time differentiation yields,

(20) $$\begin{array}{@{}rcl@{}} \frac{d(x,t_{i+1})-d(x,t_{i})}{t_{i+1}-t_{i}}&=&v(x,t_{i})|\nabla d(x,t_{i})|,\\ \frac{d(x,t_{i})-d(x,t_{i-1})}{t_{i}-t_{i-1}}&=&v(x,t_{i})|\nabla d(x,t_{i})|. \end{array} $$

The last equation can be directly used to extrapolate the essential support essup{*u*(*x*,*t*_*i*+1_)}, provided the probability density *u*(*x*,*t*) is known in time points *t*_*i*−1_ and *t*_*i*_,

(21) $${} \begin{aligned} &S' = D^{-1}\left(\vphantom{(t_{i+1}-t_{i})\frac{D\, \text{essup}\{u(x,t_{i})\} - D\, \text{essup}\{u(x,t_{i-1})\}}{t_{i}-t_{i-1}}}D\, \text{essup}\{u(x,t_{i})\}\right. \\ &\quad\,\,\, +\left.(t_{i+1}-t_{i})\frac{D\, \text{essup}\{u(x,t_{i})\} - D\, \text{essup}\{u(x,t_{i-1})\}}{t_{i}-t_{i-1}} \right)\!. \end{aligned}  $$

The estimation provides a possibility to approximate the actual density *u*(*x*,*t*_*i*+1_) with the numerical scheme (7-10) involving nodes exclusively from

(22) $$ S:=D^{-1}\left(D\, \left(\text{essup}\{u(x,t_{i})\}\bigcup S'\right) -\gamma\right).  $$

Here the parameter *γ*>0 extends the subdomain as there should be a sufficient layer of basis functions around the essential support. This is necessary to interpolate the density *u*(*x*,*t*) on the boundary. Having the approximation $\tilde {u}(x,t_{i+1})$ in turn permits to compute $\text {essup}\{\tilde {u}(x,t_{i+1})\}$ and to evaluate the reliability of the prior estimate *S*^′^. On the basis of this information we can accept results at *t*_*i*+1_ or decide to use a smaller time step.

The complete numerical strategy shapes as a sequence of the following steps:

- compute the essential support *S*_0_=essup{*u*_0_(*x*)} for the initial condition *u*_0_(*x*); choose a system of basis functions with centres *x*^*i*^∈*D*^−1^(*DS*_0_−*γ*) that provides a sufficient approximation to $\|\tilde u_{0}(x)-u_{0}(x)\|<p_{\text {threshold}}$;
- using (10) perform integration of the approximation to *u*(*x*,*t*) on a small interval [0,*t*_1_] and compute the new essential support *S*_1_; set *i*=1;
- if *t*_*i*_<*t*_*end*_, choose *t*_*i*+1_=*t*_*i*_+*h*; using (22), extrapolate the value for *S*_*i*+1_ utilising (22) and compute the corresponding basis; integrate the system up to *t*_*i*+1_; validate *S*_*i*+1_ by computing $\|D(\text {essup}\,{\tilde u(x, t_{i+1})}) -D(S_{i+1})\|$; in the case of satisfactory choice for *S*_*i*+1_, increase *i* by one and repeat the step, otherwise repeat the step with a smaller value for *h*.

Here, essential support threshold *p*_threshold_, initial time step *h*, and density of the basis coverage *γ* are parameters of the method. The parameter of *α*-hull has grid step *h* as the lower bound and is chosen to be 2*h* in the numerical examples that follow.

## Discussion

### Self-regulating gene

One of the simplest models of a gene regulatory network consists of a single gene regulated by a self-generated proteomic atmosphere. In this model the gene is represented by a master equation governing evolution of two probability distributions: *u*_on_(*x*,*t*) corresponding to situations when the DNA is free (on state), *u*_off_(*x*,*t*) corresponds to cases when DNA has a repressing protein bound to it (off state). Here *x* denotes copy numbers of proteins, *t* represents time. The master equations for the self-regulating gene model read as [33],

(23) $$ \begin{aligned} \frac{\partial}{\partial t}u_{\text{on}}(x,t)&=g_{\text{on}} (u_{\text{on}}(x-1,t)-u_{\text{on}}(x,t))\\ &\quad+ k\left((x+1)u_{\text{on}}(x+1,t)-xu_{\text{on}}(x,t)\right) \\ &\quad- h(x)u_{\text{on}}(x,t)+ fu_{\text{off}} (x,t),\;\;x>0;\\ \frac{\partial }{\partial t}u_{\text{on}}(x,t)&=-\,g_{\text{on}}u_{\text{on}}(x,t)+k(u_{\text{on}}(x+1,t)\\ &\quad +u_{\text{off}}(x+1,t)),\;\; x=0;\\ \frac{\partial }{\partial t}u_{\text{off}}(x,t)&=g_{\text{off}} (u_{\text{off}}(x-1,t)-u_{\text{off}}(x,t))\\ &\quad+ k\left((x+1)u_{\text{off}}(x+1,t)-xu_{\text{off}}(x,t)\right) \\ &\quad+h(x)u_{\text{off}}(x,t)- fu_{\text{on}}(x,t),\;\;x>1;\\ \frac{\partial }{\partial t}u_{\text{off}}(x,t)&=-\,g_{\text{off}}u_{\text{off}}(x,t)+k\left(2u_{\text{off}}(x+1,t)\right. \\ &\left.\quad-u_{\text{off}}(x,t)\right)+ h(x)u_{\text{on}}(x,t)\\ &\quad\hspace{.5pt}-fu_{\text{off}}(x,t),\;\; x=1. \end{aligned}  $$

Here, *g*_on_,*g*_off_ are rates of protein production in the free and bound states, *k* is a rate of protein degradation, and *f* is the rate of the repressor protein releasing from the repressor site. In case of monomers, the net binding rate is simply proportional to the number of proteins, *h*(*x*)=*hx*. In a biologically more relevant case - the dimerisation upon binding, the rate is defined as *h*(*x*)=*h*/2*x*(*x*−1) [34]. The master equations presented in (23) can be reformulated in the operator form (6),

(24) $${} \begin{aligned} \frac{\partial}{\partial t}u_{\text{on}}(x,t)&=g_{\text{on}} (T_{1} -I)u_{\text{on}}(x,t)\\ &\quad+ k (T^{-}_{1}-I)W_{x} u_{\text{on}}(x,t) -W_{h(x)}u_{\text{on}}(x,t)\\ &\quad+ W_{f}u_{\text{off}}\,(x,t) +kW_{x=0}u_{\text{off}}\,(x+1,t);\\ \frac{\partial }{\partial t}u_{\text{off}}\,(x,t)&\!=g_{\text{off}}\, (T_{1} \!-I)u_{\text{on}}(x,t)+\! k (T^{-}_{1}\!-I)W_{x} u_{\text{off}}\,(x,t)\\ &\quad + W_{h(x)}u_{\text{off}}\,(x,t)- W_{f}u_{\text{on}}\ (x,t).\\ \end{aligned}  $$

Here *W*_*x*=0_ acts as unity operator for *x*=0 and zero operator for *x*>0. The values of the probability distributions that contradict the physical nature of the process are defined to vanish, that is *u*_on_(*x*,*t*)=0,*x*<0, and *u*_off_ (*x*,*t*)=0,*x*<1. Since extra terms are present, i.e *W*_*h*(*x*)_, this important system of two one-dimensional master equations does not fall into the class of CMEs; nevertheless it can be discretized by the proposed numerical toolbox. In order to compare the numerical results with the previous findings it is convenient introduce the following unitless parameters:

$$X^{ad} = \frac{\left(g_{\text{on}}+g_{\text{off}}\right)}{2k};\;\; X^{eq} = \frac{f}{h}; \;\; \omega = \frac{f}{k}. $$

**Fig. 2:**
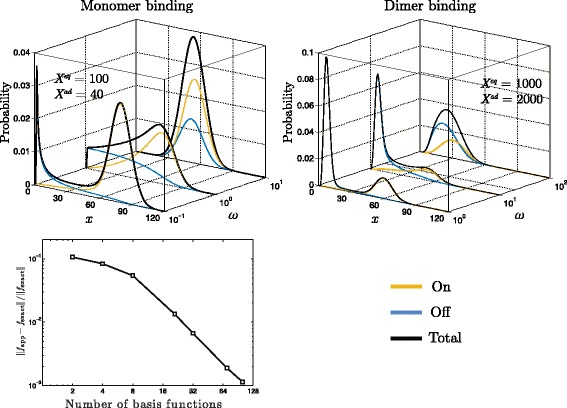
Self-regulating gene model. Top panels depict probabilities of gene expression as a function of protein number, *x*, obtained for monomer and dimer binding cases and various values of *ω*. The bottom panel depicts convergence of the error as a function of approximation parameter - number of basis functions

As can be concluded form the stationary solutions presented in the top panels in Fig. 2, small *ω* provokes emergence of two distinct peaks in the overall probability distribution, *g*_on_+*g*_off_, for both cases: monomer and dimer binding. One peak corresponds to the repressed protein production, the other to a much higher protein production (due to free DNA). As protein binding/unbinding becomes faster (i.e. augmented *ω*) the peaks tend to fuse. In the case of monomer binding the exact analytical solution is known [33]. This gives the possibility to test the method’s convergence on the self-regulatory protein problem. The convergence diagram of relative error of the approximation measured in the *l*_2_-norm is presented in the lower panel in Fig. 2. In this example the approximation grid has been kept constant due to a small number of system states. As will be shown in the next case studies a much bigger computational challenge can be encountered when treating problems of dimensionality greater then one.

### Bistable toggle switch

A classical example of a bistable genetic toggle switch has been both, a comprehensive model explaining experimental data, for instance on E. coli as in Ref. [25], and a challenging test for various numerical methods to solve the CME [8, 12, 35, 36]. In the context of the current paper, we consider the model for its peculiar tendency to form probability landscapes with two maxima. The model describes a pair of mutually repressing genes *A* and *B*. Each of the species inhibits the production of the competing repressor by binding to the corresponding genetic control sequences of the promoter. If *A* becomes abundant the production of *B* is inhibited and the system is in a stable state of high *A* and low *B*. If due to stochastic fluctuations the amount of *A* decreases or the amount of *B* is sufficiently high, the switch might flip and *B* becomes abundant and *A* repressed. More formally, at every point in time the state of the system is characterised by a 2-dimensional vector (*x*,*y*), where *x* represents the copy numbers of *A* and *y* represents the copy numbers of *B*. The model consist of the following reaction channels,

(25) $$ \begin{aligned} R_{1}: (x,y) \xrightarrow{a_{1}(x,y)} ({x}+1,y); \\ R_{2}: (x,y) \xrightarrow{a_{2}(x,y)} ({x}-1,{y}); \\ R_{3}: (x,y) \xrightarrow{a_{3}(x,y)} ({x},{y}+1); \\ R_{4}: (x,y) \xrightarrow{a_{4}(x,y)} ({x},{y}-1); \end{aligned}  $$

where the propensities are defined as follows,

(26) $$ \begin{aligned} a_{1}(x,y) = & \frac{c_{1}}{ c_{2} + y^{\beta}}; & a_{2}(x,y) = & \ c_{3} x ; \\ a_{3}(x,y) = & \frac{c_{4}}{ c_{5} + x^{\gamma}}; & a_{4}(x,y) = & \ c_{6} y. \end{aligned}  $$

Let’s denote *u*(*x*,*y*,*t*) the probability of the system to be in a state (*x*,*y*) at time *t*. Rephrasing mechanisms (25) in terms of the CME presented in the operator form (6) yields

(27) $$ \begin{aligned} \frac{\partial }{\partial t}u(x,y,t)&=(T_{1}-I) W_{a_{1}(x,y)}+ (T_{1}^{-}-I) W_{a_{2}(x,y)}\\ &\quad +(T_{2}-I) W_{a_{3}(x,y)}+(T_{2}^{-}-I) W_{a_{4}(x,y)},\\ u(x,y,0)&=u_{0}(x,y). \end{aligned}  $$

**Fig. 3:**
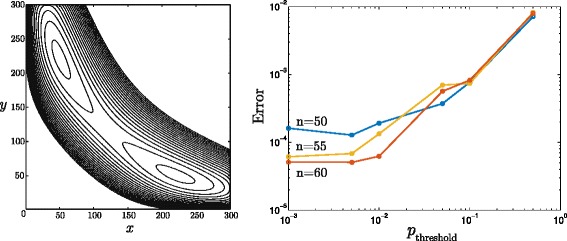
Exact steady-state solution to the bistable toggle switch problem and convergence diagram for approximations (*Left:*) each level line corresponds to one order of magnitude of the exact steady state solution; in total, 20 level lines presented. (*Right:*) approximation error plotted versus the cutoff parameter *p*_threshold_ for various numbers of basis functions, *n*

The probability distribution that solves the CME (25) equipped with the parameter set

(28) $$ \begin{aligned} c_{1} = & \ c_{4} = 3 \cdot 10^{3}, \\ c_{2} = & \ c_{5} = 1.1 \cdot 10^{4}, \\ c_{3} = & \ c_{6} = 10^{-3}, \\ \beta=& \ \gamma=2, \end{aligned}  $$

is known to develop two peaks that correspond to the two semi-stable states [12, 36]. In principle, Eq. (27) can be considered on the state space (*x*,*y*)∈ [ 0,300]× [ 0,300] and integrated in time up to a very high precision by numerical exponentiation. This approach, however, employs matrices of a size 90601×90601, and is far from being optimal if an objective is to recover only probabilities that are significantly greater than zero. The stationary solution *u*(*x*,*y*), depicted in Fig. 3a, demonstrates that the essential support corresponding to *p*_threshold_=0.01· max*u*(*x*,*y*) occupies only a small fraction of states.

**Fig. 4:**
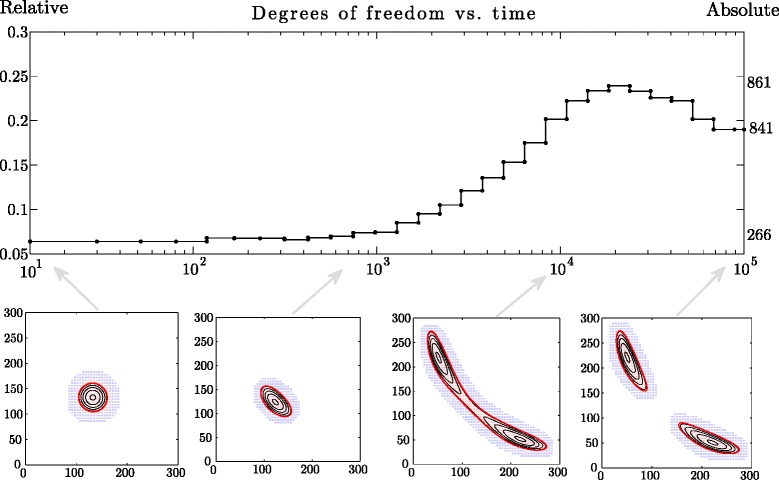
Numerical solution to the bistable toggle switch problem with splitting topology. The threshold parameter *p*_threshold_ has been chosen to force the essential support evolve from a simple connected into a two-connected domain: (*top*) degrees of freedom (vertical axis) as a function of time (horizontal axis); (*bottom*) four panels represent the solution on various time stages: the initial conditions; solution shortly after the start; solution just before the split; and the final, steady state distribution supported on a two-connected domain

In contrast to the full-state-space approach, the numerical algorithm proposed in the previous section employs a much smaller number of the degrees of freedom. Let the approximation basis consist of radial basis functions (7) centred in grid points *x*^*i*^∈ [0,*h*,2*h*,…300]^2^, where *h* is the grid step. Running the algorithm starting with initial condition

(29) $$ u_{0}(x,y)=e^{-\frac{(x-133)^{2}+(y-133)^{2}}{266}},\quad (x,y)\in\Omega,  $$

provides us with a succession of domains that support only the part of the solution that has values greater than *p*_threshold_. As illustrated in the bottom panels of Fig. 4, the threshold can be chosen so as to make the final essential support two-connected (see also Additional file 1). The degrees of freedom required for the parametrisation of the solution remain relatively small; the number gradually increases as the distribution progresses from its initial state, but decreases after the essential support evolves into a two-connected domain, see the top panel in Fig. 4. Another example regards asymmetrical initial conditions

(30) $$ u_{0}(x,y)=e^{-\frac{(x-133)^{2}+y^{2}}{266}},\quad (x,y)\in\Omega,  $$

**Fig. 5:**
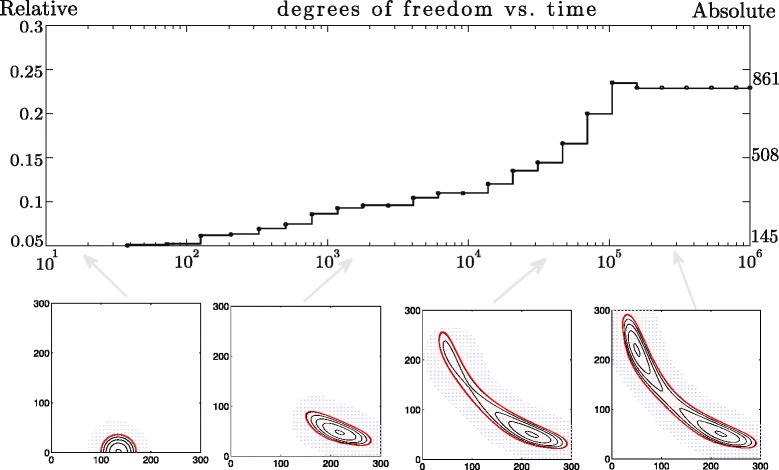
Numerical solution to the bistable toggle switch problem with asymmetrical initial conditions. (*top*) Relative degrees of freedom (vertical axis) as a function of time (horizontal axis); (*bottom*) four panels representing the solution on various stages: asymmetric initial conditions (*x*_1_=133, *x*_2_=0,); unimodal distribution; bimodal asymmetric distribution; the steady state resulting in a symmetric bimodal structure

Such initial conditions force the distribution to evolve in time into one mode of the bistable solution at first, and eventually, equilibrating into the bistable solution as depicted in Fig. 5. In this case setting *p*_threshold_ to a large value might lead to discovering only one mode of the solution. Therefore, it is important to ensure the deviation between total probabilities of the initial conditions and the approximate solution is kept small,

$$\left(\sum\limits_{x,y} u_{0}(x,y,t) - \sum\limits_{x,y} u(x,u,t)\right) / \sum\limits_{x,y} u_{0}(x,y,t) <\epsilon. $$

Shall the deviation increase over a certain level, the value of *p*_threshold_ should be lowered. As depicted in the right panel of Fig. 3, both *p*_*threshold*_ and number of basis functions, *n*, have a direct influence on the error of the approximation. As can be seen in Fig. 3, decreasing the value of *p*_*threshold*_ lowers the approximation error up to a certain saturation point. The further improvements are possible only by increasing the number of basis functions.

#### Comparison with Gillespie SSA

**Fig. 6:**
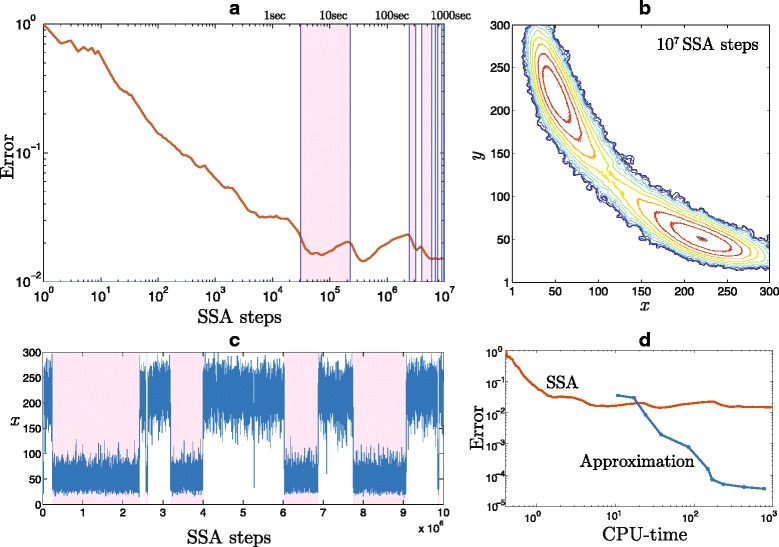
Probability distribution via Gillespie SSA **a** Error of the probability distribution obtained form a SSA-generated trajectory is plotted versus number of steps. The upper axis indicates cpu-time. The vertical bands represent switching of the two-stable system. **b** The level lines of the probability distribution recovered by analysing frequency of a single SSA trajectory visiting each of the states followed by smoothing with a rectangular 3×3 window. **c** *x*-coordinate of a single SSA trajectory plotted versus number of steps demonstrates the switching nature of the stochastic system. **d** Error as a function of CPU-time for the SSA and the approximation

Since the proposed method deals with a CME, that produces a complete probability distribution, it cannot be thought as an alternative to Monte Carlo algorithms, which simulate a single trajectory of a stochastic process. That said, it is possible to extract the frequency of visiting each of the system states by a stochastic process (e.g. generated by Gillespie SSA), and eventually relate it asymptotically to the probability distribution providing the trajectory is long. On practice, this might be a formidable task: producing a long enough trajectory may consume considerable cpu-time, even more, in cases of multi stability the convergence of the SSA may be hard to estimate. For example, an error diagram of the probability distribution extracted from SSA simulations for the toggle switch problem is given in Fig. 6a. Initially, the error decreases with increasing number of SSA steps, but as soon as the system switches the mode for the first time (as indicated by the vertical bars), the error decrease slows down considerably. The result extracted form a trajectory with 10^7^ SSA steps deviates form the exact solution by 0.02 in *l*_2_ norm, and even after being smoothed out by a rectangular 3×3 window the level lines of the probability distribution contain considerable artefacts, see Fig. 6b. The trajectory itself is depicted in Fig. 6c. The probability distribution obtained by the approximation on Gaussian basis functions with interface tracking demonstrates a much better accuracy for even lower cpu-time. As can be seen in Fig. 6d, the error of two magnitudes lower then the one provided by the SSA is obtained for the same cpu-time, 200 sec. It is important to note that comparison of cpu-time might be weakly biased by a particular implementation of the algorithms, that have such a different nature.

### Tristable toggle switch

Analogously to the bistable toggle switch a theoretical tristable system describing three mutually competing species *A*,*B*,*C* is considered. In this case the state of the system is characterised by three copy numbers (*x*,*y*,*z*), and the model consist of the following reactions channels,

(31) $$ \begin{aligned} R_{1}: (x,y,z) \xrightarrow{a_{1}(x,y,z)} ({x+1},y,z),\\ R_{2}: (x,y,z) \xrightarrow{a_{2}(x,y,z)} ({x-1},{y},z), \\ R_{3}: (x,y,z) \xrightarrow{a_{3}(x,y,z)} ({x},{y+1},z), \\ R_{4}: (x,y,z) \xrightarrow{a_{4}(x,y,z)} ({x},{y-1},z), \\ R_{5}: (x,y,z) \xrightarrow{a_{5}(x,y,z)} ({x},{y},{z+1}), \\ R_{6}: (x,y,z) \xrightarrow{a_{6}(x,y,z)} ({x},{y},{z-1}), \\ \end{aligned}  $$

where the propensities are defined as follows,

(32) $$ \begin{aligned} a_{1}(x,y,z)=&\ \frac{c_{1}}{c_{2}+(y+z)^{\beta}}, & a_{2}(x,y,z)=&\ c_{3} x, \\ a_{3}(x,y,z)=&\ \frac{c_{4}}{c_{5}+(x+z)^{\gamma}}, & a_{4}(x,y,z)=&\ c_{6} y,\\ a_{5}(x,y,z)=&\ \frac{c_{7}}{c_{8}+(x+y)^{\zeta}}, & a_{6}(x,y,z)=&\ c_{9} z. \end{aligned}  $$

The distribution *u*(*x*,*y*,*z*,*t*) associates a probability to each system state defined by the vector of copy numbers (*x*,*y*,*z*) at time *t*. The distribution obeys the following CME,

(33) $$ {}\begin{aligned} \frac{\partial }{\partial t}u(x,y,z,t)&=(T_{1}-I)W_{a_{1}(x,y,z)} +(T_{1}^{-}-I)W_{a_{2}(x,y,z)}\\ &\quad+(T_{2}-I)W_{a_{3}(x,y,z)} + (T_{2}^{-}-I)W_{a_{4}(x,y,z)} \\ &\quad+(T_{3}-I)W_{a_{5}(x,y,z)} + (T_{3}^{-}-I)W_{a_{6}(x,y,z)}, \\ u(x,y,z,0)&=u_{0}(x,y,z). \end{aligned}  $$

Similarly to the previous example, we employ a parameter set that yields a symmetric solution,

(34) $$ \begin{aligned} c_{1}=&\ c_{4}=c_{7}=3\cdot 10^{3} \\ c_{2}=&\ c_{5}=c_{8}=1.1\cdot10^{4},\\ c_{3}=&\ c_{6}=c_{9}=10^{-3}, \\ \beta=&\ \gamma=\zeta=2. \end{aligned}  $$

**Fig. 7:**
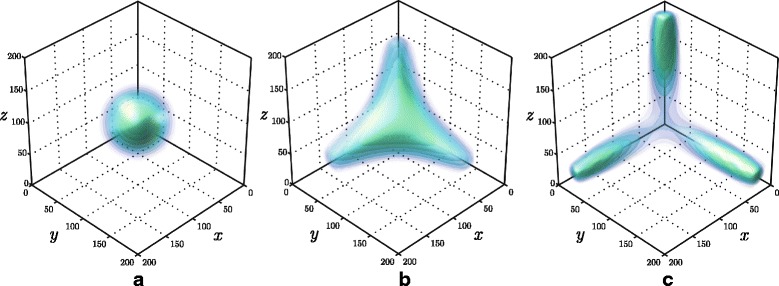
Solution to the tristable toggle switch problem. The panels illustrate isosurfaces of three consecutive time-stages of the solution: **a** initial conditions, unimodal distribution; **b** intermediate distribution; **c** the steady state, a trimodal distribution

**Fig. 8:**
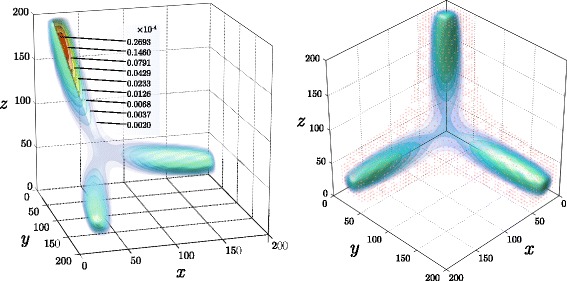
The steady state solution to symmetrical tristable toggle switch. (*left*) a part of the distribution is removed to reveal the inner structure of the isosurfaces. (*right*)The final discretisation mesh is highlighted

The stationary solution to Eq. (33) constitutes an interesting example of how complex the essential support might be. Indeed, as can be seen in Fig. 7, depending on the value of the significance level *p*_threshold_, the three dimensional domain is exhibiting various types of topology: it is non-convex, either simple connected or three-connected. In both cases a highly adaptive parametrisation is essential for saving computational resources. Fig. 8 shows the inner structure of enclosed isosurfaces on the left panel, while the basis function centres used for the stationary solution are presented on the right panel. See also Additional file 2.

### Stem cell differentiation problem

**Fig. 9:**
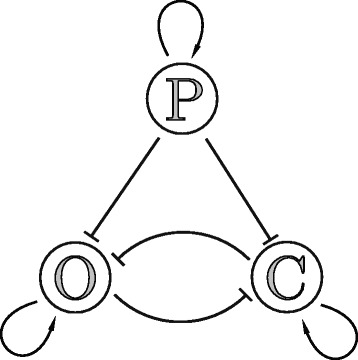
The conceptual model for the osteochondro switch. Interactions between progenitor maintenance factor (P) osteogenic (O), and chondrogenic (C) TRs are depicted with arrows: activation is denoted with a sharp arrow →, inhibition is denoted with a stump arrow ⊣

Let us consider the osteochondro switch (OCS) model introduced by Schittler et al. [29]. The model represents a particular example of two mutually inhibiting key transcriptional regulators (TRs), which determine the cell fate of osteochondro progenitor cells, see Fig. 9. The system state is represented by the three state variables *x*,*y*,*z*, corresponding to the progenitor (*z*), osteogenic (*x*), and chondrogenic (*y*) TRs. Relating to experimental data, these would be (a rough measure) of mRNA levels, or a more precise measure of transcription factor activated from reporter genes [29].

In stimuli-free setup Schittler et al. considered the set of ordinary differential equations

(35) $$ \left\{ \begin{array}{l} x'(t)=\frac{a_{o} x^{\beta}(t) +b_{o}}{m_{o}+c_{oo}x^{\beta}(t)+c_{oc} y^{\beta}(t)+ c_{op}z^{\beta}(t)}-k_{o} x(t),\\ y'(t)=\frac{a_{c} y^{\beta}(t) +b_{c}}{m_{c}+c_{cc}y^{\beta}(t)+c_{co} x^{\beta}(t)+ c_{cp}z^{\beta}(t)}-k_{c} y(t),\\ z'(t)=\frac{a_{p} z^{\beta}(t) +b_{p}}{m_{p}+c_{pp}z^{\beta}(t)}-k_{p} z(t),\\ \end{array} \right.  $$

where the meaning and values of the coefficients are given in Table 1. A detailed bifurcation analyses of (35) performed in [29] reveals that depending on the parameter set the system may operate in bistable or tristable regimes. The solution to the differential equation system (35), however, does not provide an appropriate description since a single trajectory can only converge to one of the stationary states, whereas in practice, the system is flipping between stable states due to chemical or thermal noise. Hence, the switching behaviour can only be reproduced employing a distributional description of the system state.

**Table 1:** Parameter set used for simulations of the cell differentiation model, as suggested in Ref. [29]

Cell type	Parameter	Value	Description
*	*β*	2	Hill coefficient
Progenitor, (*P*)	*a*_*p*_	0.2	Auto-activation
	*b*_*p*_	0.5	Basal activity
	*m*_*p*_	{8, 10}	Inflection point
	*c*_*pp*_	0.1	Self-inhibition strength
	*c*_*op*_, *c*_*cp*_	0.5	Inhibition strength on *x*_*o*_,*x*_*c*_
	*k*_*p*_	0.1	Decay rate
Osteoblast and Chrondrocyte, (*O*,*C*)	*a*_*o*_, *a*_*c*_	0.1	Autoactivation
	*b*_*o*_, *b*_*c*_	1	Basal activity
	*m*_*o*_, *m*_*c*_	1	Inflection point
	*c*_*oo*_, *c*_*cc*_	0.1	Self-inhibition strength
	*c*_*oc*_, *c*_*co*_	0.1	Mutual inhibition strength
	*k*_*o*_, *k*_*c*_	0.1	Decay rate

Rewriting the cell differentiation mechanism expressed in (35) in terms of the CME yields the same differential equations as in (33) equipped with a specific set of asymmetric propensities *a*_*i*_(*x*,*y*,*z*),

(36) $${} {\fontsize{8.4pt}{12.6pt}\selectfont{\begin{aligned} a_{1}(x,y,z)=&\ \frac{a_{o} x^{\beta}(t) +b_{o}}{m_{o}+c_{oo}x^{\beta}(t)+c_{oc} y^{\beta}(t)+ c_{op}z^{\beta}(t)}; &\!\!\! a_{2}(x,y,z)=&\ k_{o} x ; \\ a_{3}(x,y,z)=&\ \frac{a_{c} y^{\beta}(t) +b_{c}}{m_{c}+c_{cc}y^{\beta}(t)+c_{co} x^{\beta}(t)+ c_{cp}z^{\beta}(t)}; &\!\!\! a_{4}(x,y,z)=&\ k_{c} y;\\ a_{5}(x,y,z)=&\ \frac{a_{p} z^{\beta}(t) +b_{p}}{m_{p}+c_{pp}z^{\beta}(t)}; &\!\!\! a_{6}(x,y,z)=&\ k_{p} z. \end{aligned}}}  $$

**Fig. 10:**
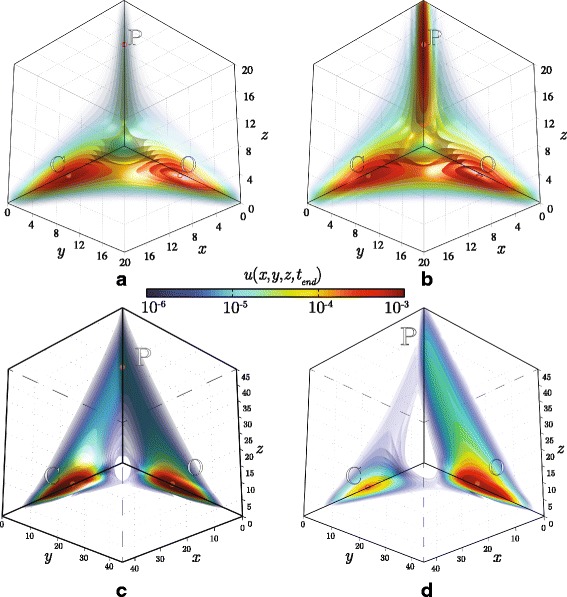
Three-dimensional steady state solutions to the cell differentiation model obtained for various parameter sets **a** two-stable mode corresponds to the default parameter set. **b** three-stable mode corresponds to progenitor inflection point *m*_*p*_=8. **c**, **d** Scaling up auto-activation and basal activity parameters *a*_*p*,*o*,*c*_, *b*_*p*,*o*,*c*_ by a factor 2.5 leads to stronger separation of chrondrocyte and osteoblast states. **d** Pro-osteoblast stimulus promotes *P*→*O* transition

Equation 33 is linear, hence any initial condition leads to a unique stationary solution. Various parametric sets for the OCS model may yield solutions with very different essential supports. This is demonstrated in Fig. 10 where isosurfaces of the three-dimentional probability distribution *u*(*x*,*y*,*z*,*t*_*end*_) are presented (see also Additional file 3). A bistable solution corresponding to the basic parameter set as used in [29] is depicted in Fig. 10a. Choosing a smaller value for the inflection point *m*_*p*_=8 forces the solution to become tristable, Fig. 10b. Scaling up the auto-activation and basal activity parameters *a*_*p*,*o*,*c*_, *b*_*p*,*o*,*c*_ by a factor 2.5 produces more segregated maxima in the solution, additionally, the absolute values of the copy numbers, *x*,*y*,*z*, are higher as depicted in Fig. 10c. Finally, an effect of biased differentiation is modelled by modifying the Hill function associated with osteogenic cells to include a pro-osteogenic stimulus *z*_*o*_>0,

$$a'_{1}(x,y,z)= \frac{a_{o} x^{\beta}(t) +b_{o}+z_{o}}{m_{o}+c_{oo}x^{\beta}(t)+c_{oc} y^{\beta}(t)+ c_{op}z^{\beta}(t)}. $$

**Fig. 11:**
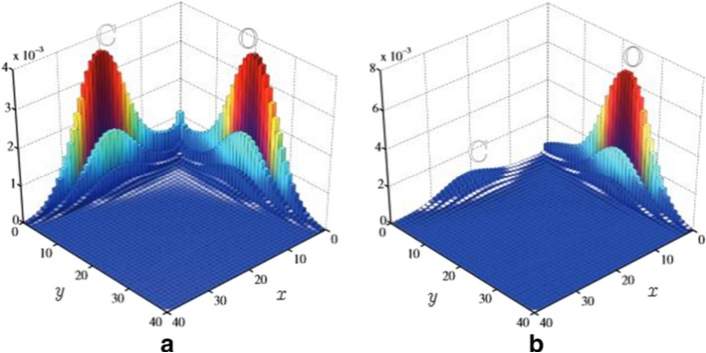
Steady-state probability distributions defined over *x*,*y*- plane demonstrates the effect of the pro-osteoblast stimuli. **a** No stimulus case corresponds to a symmetrical solution. **b** Application of the pro-osteoblast stimulus leads to an asymmetrical solution with a clear domination of osteoblast states

Figure 10d illustrates the three dimensional probability density corresponding to stimulus *z*_*o*_=6; the remaining parameters are identical to those in Fig. 10c. An effect of pro-osteogenic stimuli is further explored in Fig. 11, where the two-dimensional marginal distribution $\sum \limits _{z}u(x,y,z,t_{\textit {end}})$ is plotted for two cases: *z*_*o*_=0 (panel a), and *z*_*o*_=6 (panel b).

**Fig. 12:**
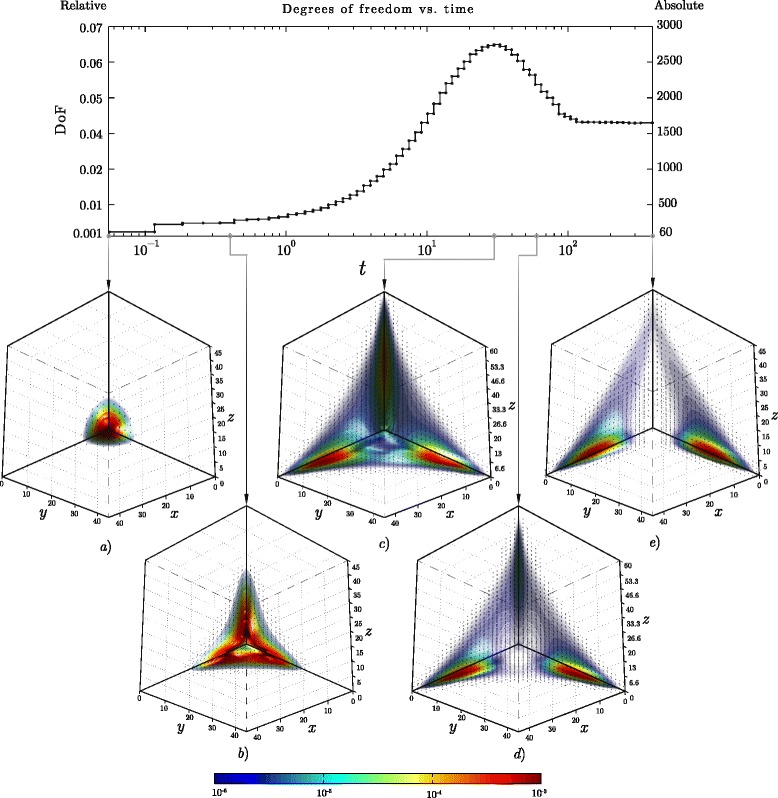
Time evolution of the solution to the osteochondro switch problem. Model parameters have been chosen so the essential support of the three-dimensional distribution progressively expands at early times, but eventually shrinks before reaching the steady-state, forcing the degrees of freedom of the approximation scheme to decrease

When considering the time evolution of the solution to the OCS problem, it is natural to expect that the number of degrees of freedom required by the approximation scheme is not constant. For instance, if the simulation for the case study presented in Fig. 10c is started with the initial condition

$$u_{0}(x,y,z)=e^{-\frac{(x-1)^{2}+(y-1)^{2}+(z-1)^{2}}{6}}, $$

the essential support undergoes complex transformation before the solution reaches the stationary solution, as depicted in Fig. 12. The top panel of Fig. 12 shows degrees of freedom as a function of time. Even though the degrees of freedom increase initially, reach maximum, and decrease before the system arrives to the steady state, only a small fraction of the full grid nodes is employed at each point of time. In fact, the maximum number of approximation parameters, 2701, is only 7 *%* of the overall grid nodes, and 1 *%* of the total number of states, assuming *x*,*y*,*z*<60. In the previous case study, these values are even more dramatic: at most 3887 basis functions were used for the approximations, which is 2 *%* of the full grid and 0.05 % of the total number of states assuming the upper bound *x*,*y*,*z*<200.

## Conclusions

We proposed a numerical method for the approximation of the solution to a wide range of CME based problems that have been hard to tackle until now. The fact that the method recovers a full representation of multi-dimensional distributions makes it especially attractive for cases of multi-stability.

In order to reduce the amount of computational resources, the unknown distribution is searched as a linear combination of Gaussian radial basis functions. The efficiency of the method is improved even further by predicting a manifold containing states with probabilities that are greater than a certain significance threshold in every time step. The prediction is done on the basis of information available form previous time steps. It allows to keep the degrees of freedom of the approximation very close to the optimal value corresponding to the significance threshold.

The method has been applied to the following examples of two- and three-dimensional CMEs leading to multi-stable solutions:

- A bistable genetic toggle switch describing two competing species. This problem constitutes an important case: when the normal distribution is taken as initial condition, the manifold containing highly probable states undergoes drastic transformations. Its topology transits from simple connected to a two-connected domain. Since the exact solution is known for this problem, the approximation error can be evaluated.
- A tristable toggle switch, yielding a three-dimensional symmetric solution, is introduced as a generalisation of the previous problem. Although this case demonstrates a possible mechanism for three competing species and constitutes an interesting test for the algorithm, it remains a theoretical problem.
- A cell differentiation model, describing cell fate determination of osteochondro progenitor cells. The model considers two final cell types, osteoblast and chondrocytes, and is a special case of the previous example. It has been shown how variations of some important parameters affect the stationary solution. It has also been studied how a pro-osteogenic stimulus leads to a non-symmetrical solution.

Besides CME, the method has been additionally applied to a system of master equations describing a self-regulatory gene.

We expect that the method can be applied to other CME problems including those that have no a priori information available on the shape, location, or upper bound of the domain that contains states with significant probabilities. The domain is constructed and tracked in time using ideas from level set methods. The advantage of the level set approach is that one can perform numerical computations involving surfaces on a fixed Cartesian grid without having to parameterise these objects. In addition, the level set method makes it very easy to follow shapes that change topology, for example when a shape splits into two, develops holes, or the reverse of these operations.

Although the method features many advantages for multi-stable systems or systems where rare events are important, high-dimensional cases (d >4) are hard to tackle with the current implementation. In future work, we plan to relax the condition that radial basis function centres are selected form a pre-defined grid in order to reach the optimal number of degrees of freedom in the approximation and extend the algorithm to high-dimensional cases.

## Additional files

Additional file 1
**An animation illustrating the usage of the numerical method for integration of the bistable toggle switch problem.** (MPEG 1352 kb)

Additional file 2
**An animation depicting time evolution of the solution to the tristable toggle switch problem.** The solution is represented by a series of isosurfaces. The set of basis function centres used for approximation of the steady state solution is also given. (MPEG 15872 kb)

Additional file 3
**An animation depicting the time evolution of the solution to cell differentiation problem with stimuli-free setup.** The solution is represented by a series of isosurfaces. The set of basis function centres used for approximation of the steady state solution is also given. (MP4 9902 kb)

## Abbreviations

CME: Chemical master equation
dCME: Discrete chemical master equation
DNA: Deoxyribonucleic acid
FSP: Finite state projection
GBF: Gaussian basis function
MC: Monte Carlo
mRNA: Messenger ribonucleic acid
MSC: Mesenchymal stem cell
OCS: Osteochondro switch
PDF: Probability density function
TR: Transcriptional regulator
SSA: Stochastic simulation algorithm
